# Association of prenatal Cleft Lip and Palate ultrasound abnormalities with copy number variants at a single Chinese tertiary center

**DOI:** 10.1186/s13052-024-01720-y

**Published:** 2024-08-21

**Authors:** Shujuan Yan, Qiuxia Yu, Hang Zhou, Ruibin Huang, You Wang, Chunling Ma, Fei Guo, Fang Fu, Ru Li, Fucheng Li, Xiangyi Jin, Li Zhen, Min Pan, Dongzhi Li, Can Liao

**Affiliations:** Prenatal Diagnostic Center, Guangzhou Women and Children’s Medical Center, Guangzhou Medical University, Guangzhou, Guangdong 510620 China

**Keywords:** Cleft lip and palate, Prenatal diagnosis, Chromosomal microarray analysis, Genetic counseling

## Abstract

**Backgroud:**

A systematic analysis was conducted to investigate the molecular etiology of fetal cleft lip and/or palate (CL/P) and the association between various types of CL/P and copy number variations (CNVs), as well as their impact on birth outcomes.

**Methods:**

In this retrospective study conducted between January 2016 and July 2022, a cohort of pregnancies diagnosed with fetal CL/P was enrolled and comprehensive clinical data for all cases were extracted from our medical record database, including demographic data about the pregnancies, ultrasound findings, results of Chromosomal microarray (CMA), as well as relevant pregnant and perinatal outcomes.

**Results:**

Among the 358 cases, 32 clinically significant variants in 29 (8.1%) fetuses with CL/P were detected by CMA. In 338 singleton pregnancies, the diagnostic yield of CMA in the context of CL/P fetuses was determined to be 7.7% (26/338). CP cases exhibited a relatively higher prevalence of pathogenic/likely pathogenic CNVs at a rate of 25% (3/12), followed by CLP cases at 8.0% (23/288). Notably, the CL group did not demonstrate any pathogenic/likely pathogenic CNV findings among the examined cases (0/38). The diagnostic rate of clinically significant variants was notably higher in the non-isolated CL/P group than in the isolated CL/P group (11/33, 33.3% vs. 15/305, 4.9%, *p* < 0.001). Within the remaining 20 twin pregnancies, three clinically significant variants (15%) were observed.

**Conclusions:**

This study provides powerful evidence supporting the efficacy of CMA as a valuable tool for facilitating the prenatal genetic diagnosis of fetal CL/P. The presence of CP and CLP in fetal cases demonstrated a relatively higher incidence of pathogenic/likely pathogenic CNVs. Moreover, when these cases were accompanied by additional ultrasound abnormalities, the likelihood of identifying diagnostic CNVs significantly increased. Conversely, cases of CL alone might not be associated with positive CNVs. The present data may significantly enhance prenatal diagnosis accuracy and facilitate informed genetic counseling for cases of fetal CL/P.

**Supplementary Information:**

The online version contains supplementary material available at 10.1186/s13052-024-01720-y.

## Introduction

Cleft lip and/or palate (CL/P) is the most common congenital craniofacial anomaly, with an incidence rate ranging from 1 to 2.2 per 1,000 live births, which is based on geographical location and ethnic characteristics [1, 2]. Among non-syndromic clefts, the CL/P incidence rate is twice higher in males when compared to females [3, 4]. Diagnosis of CL/P typically occurs during the second and third trimesters of pregnancy, facilitated by evaluation of mid-sagittal, coronal, and axial views of the fetal face and head [5]. The types of CL/P included cleft lip only (CL), cleft palate only (CP) and cleft lip and palate (CLP); these types of clefts may manifest either in isolation or as components of genetic syndromes. The majority of CL/P cases are isolated, while the presence of additional malformations alongside CL/P could suggest syndromic CL/P [6]. Its etiology is intricate and remains incompletely elucidated, but it is acknowledged that genetic and environmental factors play important roles in its manifestation. CL/P carries cosmetic and functional implications that necessitate postnatal corrective surgical procedures and comprehensive multidisciplinary care, encompassing neonatology, oral and maxillofacial surgery and psychological support. The long-term prognosis of the fetuses diagnosed with CL/P depends on the presence of genetic abnormalities and other structural abnormalities.

CMA is a high-resolution, whole-genome technique that surpasses chromosomal karyotyping in its ability to detect genetic abnormalities, encompassing aneuploidy, large segment deletions/duplications, as well as microdeletions, microduplications and loss of heterozygosity (LOH) [7]. CMA is particularly recommended for genetic analysis in cases involving fetal structural anomalies [8]. According to literature reports, CMA is able to identify clinically significant genomic alterations in approximately 6% of fetuses exhibiting abnormal ultrasound and with normal karyotype [9]. While there have been several research publications on prenatal diagnosis of fetal CL/P utilizing CMA [10, 11], the comprehensive assessment of the specific information about fetuses with CL/P through CMA is currently limited.

This study aims to systematically analyze the prenatal clinical and molecular characteristics of 358 Chinese patients diagnosed with CL/P at our center, further elucidate the molecular etiology of CL/P and investigate the potential correlation between chromosome abnormalities and the occurrence of fetal CL/P. It may be useful to enhance clinical screening and genetic counseling practices by providing additional information.

## Materials and methods

### Subjects and samples

This retrospective cohort study reviewed all prenatal cases of fetal cleft lip and/or palate diagnosed at the Prenatal Diagnosis Center, Guangzhou Women and Children’s Medical Center, Guangzhou, Guangdong, China, between January 2016 and July 2022. All cases underwent a routine ultrasound scan to assess fetal anatomy, with careful documentation of associated abnormalities. The identification of cleft lip and palate in ultrasound examinations involves the utilization of standard orthogonal views. The coronal view provides visualization of the nares and upper lip [12]. The midsagittal view captures the hard and soft palate, while the axial view reveals the maxillary alveolar ridge [13].

Pregnancies were categorized into groups based on the classification of cleft lip and/or palate (CL/P or CLP) and further subdivided into isolated CL/P or non-isolated CL/P groups, depending on the presence or absence of associated anomalies. It is noted that the classification of a combined cleft of both lip and palate may not be well-defined due to its rarity and complexity. Genetic counseling was conducted by the Maternal–Fetal Medicine team at our center to provide comprehensive information to parents of fetuses diagnosed with CL/P, which encompassed the potential risks associated with invasive surgery and the possible ramifications of the diagnostic findings.

The data utilized in this study were obtained through the consultation of electronic medical records and subsequent telephone follow-ups. Follow-up evaluations were carried out after childbirth, at 12 months, and then at 24 months postpartum. Within the cohort of 358 cases under investigation, it was observed that 11 cases underwent follow-up for a duration exceeding 12 months, whereas 347 cases were followed up for a period surpassing 24 months. The extracted information encompassed various aspects, including demographic data related to the pregnancies, indications for invasive examinations, ultrasound findings, results of CMA, and pregnancy outcomes. The assessment of pregnancy outcomes involved a combination of autopsy results, in cases of pregnancy termination, as well as telephone follow-ups and comprehensive case reviews, focusing on clinical outcomes, gestational age at birth or termination of pregnancy (TOP), neonatal sex, presence of CL/P, and presence of other abnormalities. The Ethics Committee of Guangzhou Women and Children’s Medical Center provided approval for this study, and written informed consent was obtained from all patients enrolled for invasive prenatal diagnosis.

### Chromosome microarray analysis

The extraction of genomic DNA from chorionic villi, amniocytes, and cord blood was carried out using the Qiagen DNA Blood Midi/Mini kit (Qiagen GmbH, Hilden, Germany) by the manufacturer’s protocol. Fetal samples were subjected to quantitative fluorescence polymerase chain reaction (QF-PCR, Guangzhou Darui Biotechnology Co., Ltd, Guangdong, China) to rapidly detect aneuploidies involving chromosomes 13, 18, 21, X, and Y and to exclude the possibility of maternal cell contamination. Subsequently, chromosome microarray analysis was conducted using either the CytoScan HD or 750 K array (Affymetrix), following the manufacturer’s instructions. Data analysis was performed using the Chromosome Analysis Suite software (Affymetrix) referring to the human assembly (GRCh37/hg19), which was applied for data analysis. The interpretation of CNVs adhered to the recommendations of the American College of Medical Genetics and Genomics and the Clinical Genome Resource for constitutional CNVs, which categorize all selected variants as pathogenic (P), likely pathogenic (LP), benign, likely benign, or variants of uncertain significance (VUS). The CytoScan 750 K or CytoScan HD arrays were employed to detect whole-genome CNVs, as well as identify loss of heterozygosity (LOH) and isodisomy of uniparental disomy (iso-UPD), and to detect mosaicism at levels above 30%. A more comprehensive description of the entire process can be found elsewhere [14].

### Statistical analysis

Statistical analyses were conducted using IBM SPSS Statistics version 26.0 (IBM, Armonk, NY, USA). The characteristics among the subgroups were compared using either the chi-square test or Fisher’s exact test, as appropriate. A significance level of *p* < 0.05 was adopted to determine statistical significance.

## Results

**Fig. 1:**
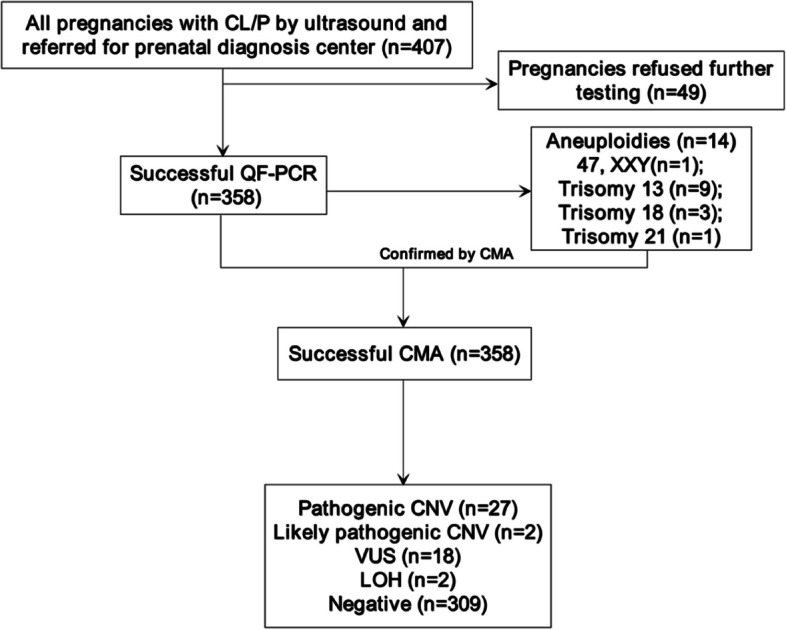
Flowchart of genetic analysis progression in cohort of fetuses with CL/P

Between January 2016 and July 2022, a total of 407 pregnancies were consulted in our center for fetal CL/P. 49 pregnancies were excluded as further testing was refused. The mean maternal age in the cohort was 30.1 (range 20.0–44.1 years). The median gestational age at which fetal anomalies were confirmed via ultrasonography was recorded at 24.4 (range 11.6–38.7 weeks), which is also the median gestational age for undergoing invasive prenatal diagnostic procedures (range 12.4–38.7 weeks). The majority of patients were diagnosed in the second trimester (277/358, 77.4%), 67 (18.7%) pregnancies were diagnosed in the last trimester, and the fewest number of patients, 3.9% (14/358), were diagnosed in the first trimester. Postnatal follow-up was accessible for 346 cases (346/358, 96.6%) and 12 (3.4%) were lost to follow-up. The flowchart of genetic analysis progression is shown in Fig. 1. Of these fetuses, 223 (64.5%) were male and 123 (35.5%) were female. The pregnancy outcome for cases with pathogenic/likely pathogenic and VUS CNVs are shown in Table 1 and Supplementary Table S1 individually. Among those 29 cases with positive CMA results, all opted for termination of the pregnancy except for one patient (case 5), whose child was prenatally diagnosed as isolated CLP alongside the identification of a likely pathogenic CNV involving a 2p16.3 deletion. Following birth, the diagnosis of CLP was confirmed, and no additional anomalies were observed during the subsequent follow-up period. In contrast, for the 18 fetuses with VUS detected by CMA, 15 cases of pregnancy outcome were obtained, including seven cases of termination of pregnancies and eight cases of live births, respectively. The pregnancy outcome for the majority of cases with a negative CMA result was full-term live births (182/295, 61.7%). Additionally, the rate of TOPs was increased in cases with pathogenic/likely pathogenic CNVs compared with a negative CMA result (28/29, 96.6% vs. 103/295, 34.9%, *p* < 0.001). In cases with negative CMA results, the rate of TOPs was obviously increased in cases with non-isolated compared with isolated CL/P (17/20 85.0% vs. 86/265, 32.5%, *p* < 0.001), in contrast to the rate of full-term live births, which was significantly decreased in cases with non-isolated compared with isolated CL/P (3/20, 15.0% vs. 179/265, 67.5%, *p* < 0.001).

**Table 1:** Clinical and chromosomal characteristics of the 29 cases with pathogenic/likely pathogenic CNVs detected

Case	MA (years)	GA (weeks)	Ultrasound Findings	CMA Results	Type of CNV	Size	Outcome
1	27.1	25.3	Isolated CP	arr18q22.3q23(70,661,548–78,013,728) × 1 arr20p13p11.23(61,662–19,077,891) × 3	Deletion Duplication	7.35 Mb 19.02 Mb	TOP
2	26.5	26.4	Isolated CLP	arr4p16.3p15.33(68,345–12,696,000) × 1	Deletion	12.63 Mb	TOP
3	31.3	30.6	Isolated CLP	arr10q26.3(131,063,320–135,426,386) × 3	Duplication	4.36 Mb	TOP
4	25.6	26.0	Isolated CLP	arr4p16.3p15.33(68,345–11,779,280) × 1	Deletion	11.71 Mb	TOP
5	31.4	23.4	Isolated CLP	^b^arr2p16.3(51,003,279–51,294,688) × 1	Deletion	291 Kb	Live birth
6^a^	35.3	27.2	Isolated CLP	arr16p11.2(29,580,021–30,190,029) × 1	Deletion	610 Kb	TOP
7	32.1	23.0	Isolated CLP	arr16p11.2(29,580,020–30,177,240) × 3	Duplication	597 Kb	TOP
8	41.3	14.0	Isolated CLP	arr(18) × 3	Duplication	77.88 Mb	TOP
9	26.6	19.6	CLP; forebrain malformation; pleural effusion; PRUV	^b^arr6p25.3p24.3(156,975–9,116,357) × 3 arr21q22.13q22.3(38,242,327–48,093,361) × 1	Duplication Deletion	8.96 Mb 9.85 Mb	TOP
10	27.0	25.6	CLP; micrognathia, CM; AVSD; biventricular enlargement	arr21q22.11(32,920,742–35,430,729) × 1	Deletion	2.51 Mb	TOP
11	39.5	12.0	CLP; NT 3.42 mm	arr12p13.33q12(173,786–34,835,837) × 2 ~ 3 mos	Duplication	38.05 Mb	TOP
12	42.6	12.1	CLP; NT 3.4 mm	arr(X) × 2	Duplication	155.1 Mb	TOP
13	32.3	14.3	CLP; absence of nasal bone; NT 4.25mmn	arr(18) × 3	Duplication	77.88 Mb	TOP
14	40.4	12.4	CLP; NT 4.32mmn	arr(13) × 3	Duplication	95.67 Mb	TOP
15	26.2	12.9	CLP; CH	arr(13) × 3	Duplication	95.67 Mb	TOP
16	34.5	28.6	CLP; renal agenesis	arr7q21.11(83,185,592–85,012,428) × 3	Duplication	1.83 Mb	TOP
17	25.7	26.0	CP; micrognathia	arr15q13.2q13.3(30,896,329–32,451,856) × 1	Deletion	1.56 Mb	TOP
18	34.6	24.0	CP; micrognathia	arr4q34.1q35.2(173,730,512–189,068,526) × 1	Deletion	15.3 Mb	TOP
19	32.7	25.4	CLP; bifid nose	^b^arr11p11.12q11(51,126,723–54,720,810) × 3	Duplication	3.59 Mb	TOP
20	27.0	25.4	CLP	arr7q32.2q36.3(130,394,280–159,119,707) × 1 arr16p11.2q11.2(31,998,168–46,463,769) × 3	Deletion Duplication	28.73 Mb 14.47 Mb	TOP
21	29.7	19.6	CLP; single atrium; CTGA	arr(13) × 3	Duplication	95.67 Mb	TOP
22	32.3	14.3	CLP; absence of nasal bone; NT 4.25 mm	arr(18) × 3	Duplication	77.88 Mb	TOP
23^a^	38.3	22.2	CLP; hypoplastic nasal bone; long bone (− 1.64 SD)	arr(21) × 3	Duplication	33.09 Mb	TOP
24	28.4	25.6	CLP; forebrain malformation; cardiac malformations	arr(13) × 3	Duplication	95.67 Mb	TOP
25^a^	28.8	24.6	CLP; forebrain malformation; CM; pyelic separation	arr(13) × 3	Duplication	95.67 Mb	TOP
26	25.6	26.4	CLP; SUA; Dandy-Walker; DORV	arr(13) × 3	Duplication	95.67 Mb	TOP
27	23.5	24.6	CLP; hydrocephalus	arr(13) × 3	Duplication	95.67 Mb	TOP
28	28.3	23.3	CLP; SUA	arr(13) × 3	Duplication	95.67 Mb	TOP
29	43.2	12.2	CLP; micrognathia; cardiac malformations; CH	arr(13) × 3	Duplication	95.67 Mb	TOP

*CLP* Cleft lip and palate, *CP* Cleft palate, *GA* Gestational age, *MA* Maternal age, *CNVs* Copy number variations, *mos* mosaicism, *TOP* Termination of pregnancy, *PRUV* Persistent right umbilical vein, *CM* Low-lying conus medullaris, *CTGA* Complete transposition of great arteries, *AVSD* Atrioventricular septal defect, *NT* Nuchal translucency, *SUA* Single umbilical artery, *DORV* Double outlet right ventricle, *CH* Cystic hygroma

^a^: one of twin fetuses, ^b^: likely pathogenic CNV

Within the cohort of 358 cases meeting the inclusion criteria, Chromosomal Microarray Analysis (CMA) identified 32 clinically significant variants in 29 cases (8.1%). Among these, three cases detected both microdeletions and microduplications (case 1, case 9 and case 20). There were 29 pathogenic copy number variants and 3 with likely pathogenic copy number variants, within which 14 were aneuploidies including XXY (*n* = 1), trisomy 13 (*n* = 9), trisomy 18 (*n* = 3), trisomy 21 (*n* = 1). There were 11 (34.4%, 11/32) with CNVs < 10 Mb, while 21 (65.6%, 21/32) were detected with CNVs > 10 Mb. The most common CNV was the 4p16.3 deletion syndrome (*n* = 2). Non-isolated CL/P was combined with the most common abnormalities combined with non-isolated CL/P were in the cardiovascular system (*n* = 10), skeletal system (*n* = 7), and multiple malformations (*n* = 10). Among the associated congenital heart defects, the most common abnormality was ventricular septal defect (*n* = 7). Table 1 shows the clinical and chromosomal characteristics of these 32 clinically significant variants in 29 cases. CMA identified 18 cases of Variants of Uncertain Significance (VUS); however, due to financial constraints, some families declined the suggestion to undergo parental CMA verification.

According to the findings presented in Table 2, the identification rate of CNVs in singleton pregnancies exhibited a higher proportion in non-isolated CL/P cases compared to isolated CL/P cases (11/33, 33.3% vs. 15/305, 4.9%, *p* < 0.001). In the context of twin pregnancies, the incidence of pathogenic CNV was found to be 15.0% (3/20), which did not demonstrate a statistically significant difference when compared to singleton pregnancies (3/20, 15.0% vs. 26/338, 7.7%, *p* = 0.433). Regarding pregnancy outcomes, the TOPs were notably higher in the non-isolated CL/P group as opposed to the isolated CL/P group (30/33, 90.9% vs. 107/305, 35.1%, *p* < 0.001). Furthermore, the rates of TOPs were observed to be higher in the CP group and CLP group in comparison to the CL group (7/12, 58.3% vs. 124/288, 40.6% vs. 5/38, 13.2%, *p* < 0.001).

**Table 2:** Stratified analysis of CNVs detection and pregnancy outcome in CL/P fetuses

Groups (Total cases)	CSVs	VUS	Live Births	TOPs	LTFU/Not LTFU
**Singleton vs. Twins (358)**					
Singleton (338)	26/338, 7.7%	17/338, 5.0%	191/338, 56.5%	137/338, 40.5%	10/338, 3.0%
Twins (20)	3/20 15.0%	1/20, 5.0%	10/20, 50.0%	8/20, 40.0%	2/20, 10.0%
* p*-value	0.433	1.000	0.569	0.068	0.140
**Isolated vs. Non-isolated**^a^** (335)**					
Isolated (305)	15/305, 4.9%	15/305, 4.9%	188/305, 61.6%	107/305, 35.1%	10/305, 3.3%
Non-isolated (33)	11/33, 33.3%	2/33, 6.1%	3/33, 9.1%	30/33, 90.9%	0/33
* p*-value	0.000	1.000	0.000	0.000	/
**Affected relatives**^a^** (335)**					
Yes (33)	1/33, 3.0%	1/33, 3.0%	18/33, 54.5%	15/33, 45.5%	0/33
No (305)	25/305, 8.2%	16/305, 5.2%	173/305, 56.7%	122/305, 40%	10/305, 3.3%
* p*-value	0.475	0.893	0.811	0.544	/
**CP/CL vs. CLP**^a^** (335)**					
CP (12)	3/12, 25.0%	1/12, 8.3%	5/12, 41.7%	7/12, 58.3%	0/12
CL (38)	0/38	2/38, 5.3%	32/38, 84.2%	5/38, 13.2%	1/38, 2.6%
CLP (288)	23/288, 8.0%	14/288, 4.9%	155/288, 53.8%	124/288, 43.1%	9/288, 3.1%
* p*-value	/	0.384	0.001	0.000	/

*CLP* Cleft lip and palate, *CL* Cleft lip, *CP* Cleft palate, *CSVs* Clinically significant variants, *VUS* Variants of unknown significance, *TOP* Termination of pregnancy, *LTFU* Lost to follow up

^a^: Comparison in singleton pregnancies

**Fig. 2:**
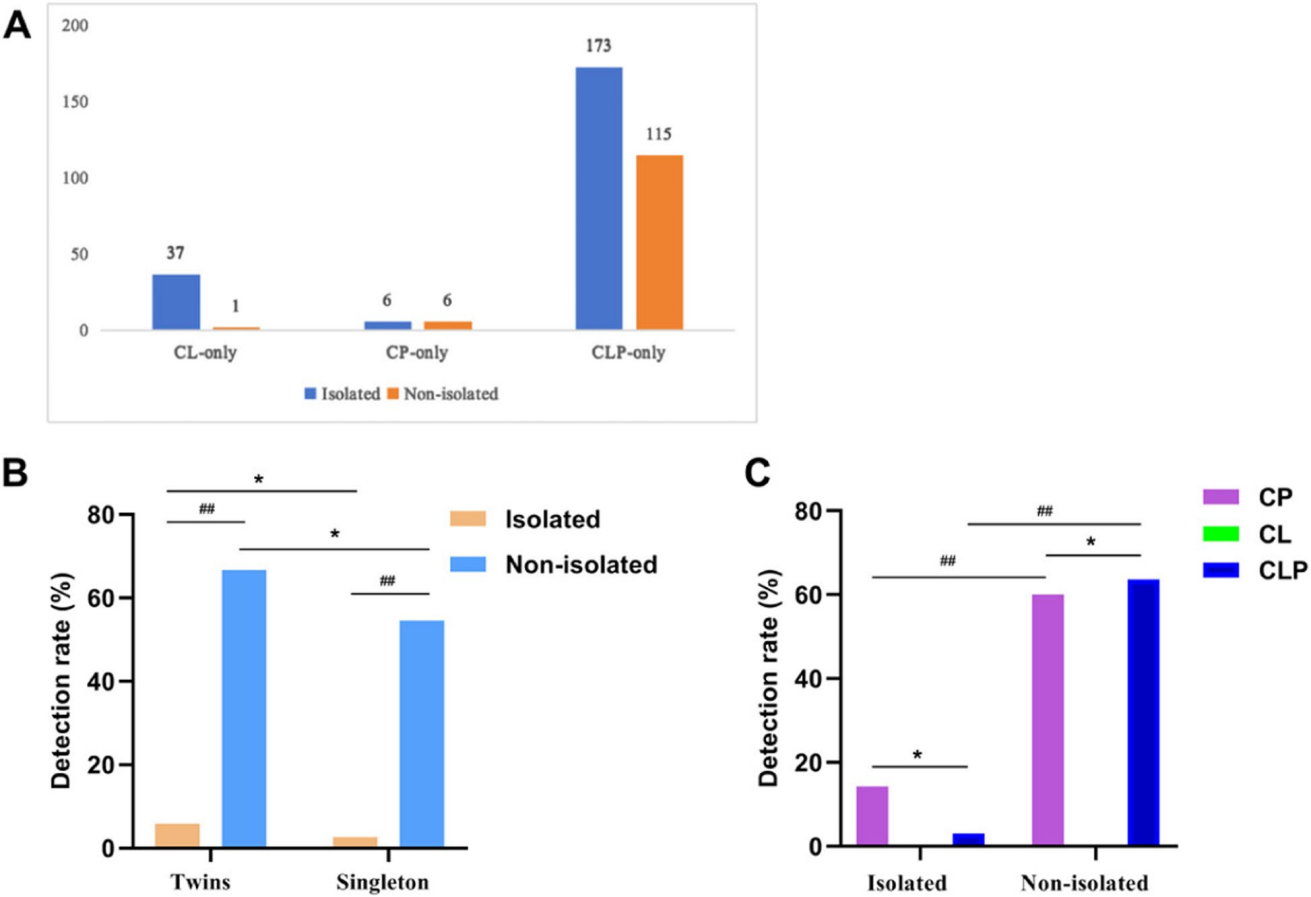
Analysis of the detection of clinically significant variants among different subgroups in singleton pregnancies affected by CL/P. **A** The number of cases with non-isolated and isolated CL/P ultrasound abnormalities that underwent CMA. **B** Comparison of CMA detection rate of isolated and non-isolated CL/P in fetuses of singleton and twin pregnancies; (**C**) Comparison of CMA detection rate of CL, CP and CLP in fetuses with isolated and non-isolated CL/P. ^*^ *p* > 0.05; ^*#*^* p* < 0.05; ^*##*^*p* < 0.001

We conducted a comparative analysis of the detection of clinically significant variants among three groups: CL, CP and CLP. Our findings revealed that the CL group did not exhibit any significant CNVs, and among the 38 cases in the CL group, 37 (97.4%) were identified with isolated CL, while only one case was classified as non-isolated. Among the 12 cases of CP, six cases were found to be associated with additional anomalies. The number of cases with non-isolated and isolated CL/P ultrasound abnormalities that underwent CMA is shown in Fig. 2A. We compared the detection rate of the CP group and the CLP group for isolated and non-isolated CL/P in singleton pregnancies intergroup and intragroup (Fig. 2B and C) and found that there was no statistical significance observed in either the intergroup and intragroup comparisons.

## Discussion

In this study, we conducted CMA on fetuses diagnosed with CL/P by ultrasound and carried out a subsequent evaluation to elucidate the genetic and clinical significance of CNVs in fetal CL/P cases. Our findings indicate that the presence of associated structural malformations in fetal CL/P is associated with a higher likelihood of clinically significant variants. The overall detection rate of clinically significant variants in our study (29/358, 8.1%) is lower compared to previous literature (40/270, 14.8%) [15]. This may be attributed to the higher proportion of isolated CL/P cases included in our study cohort compared to previous reports (305/338, 90.2% vs. 125/270, 46.3%). Isolated CL/P is a genetically complex disease with heterogeneous backgrounds. In cases where CMA produces negative results, further investigation of the underlying etiology may require the use of higher-resolution genetic tools [16], such as whole-exome sequencing (WES) or whole-genome sequencing (WGS). In our study, we identified nine cases of trisomy 13 and three cases of trisomy 18, findings that align with previous systematic reviews focused on prenatal studies revealing that trisomy 13 and trisomy 18 are the most frequently observed chromosomal abnormalities in fetuses with oral clefts. Furthermore, our study incorporates twin pregnancies and provides a comprehensive comparison between isolated and non-isolated CL/P cases, as well as between CP and CLP presentations.

CL/P can be observed in the context of other syndromic characteristics but frequently occurs as isolated. In our cohort, the majority of cases (90.2%, 305/338) presented with isolated CL/P, which cannot be attributed to a singular easily identifiable mutation; rather, they are likely influenced by a multitude of risk factors and genetic variants [17, 18]. Non-isolated CL/P cases are associated with additional abnormal morphological characteristics, often occurring as part of a syndrome. These syndromic defects are attributed to mutations that lead to the loss of function of one or more genes [19]. In our cohort, there was a significant difference in the detection rate of isolated CL/P and non-isolated CL/P (11/33, 33.3% vs. 15/305, 4.9%, *p* < 0.001), a finding which is similar to previous studies [11].

We conducted inter-group and intra-group comparisons of isolated and non-isolated CP and CLP in singleton births and found no statistically significant differences (3/12, 25.0% vs. 23/288, 8.0%, *p* = 0.251). Additionally, we compared isolated and non-isolated CL/P between twin and singleton births and similarly found no statistically significant differences (3/20, 15.0% vs. 26/338, 7.7%, *p* = 0.433). This observation underscores the decision to pursue prenatal diagnosis should not be based on the type of cleft or whether the pregnancy is singleton or twin, but rather on the presence/absence of associated anomalies during the prenatal period. In situations involving isolated CL/P unaccompanied by concurrent anomalies and producing negative results on CMA, the use of sequencing methods with higher resolution may be considered as an option.

Further analyzing of the cleft subgroups, we found that of the isolated CL/P cases with ultrasound soft markers, 38.9% (7/18) were identified with chromosomal and genomic anomalies, including aneuploidy and pathogenic CNVs. Among the 7 cases displaying concurrent ultrasound soft markers, the most commonly observed abnormalities were increased nuchal translucency (NT) and nasal bone anomalies. This suggests that soft markers may be indicative of those cases with a higher likelihood of harboring a chromosomal abnormality, thereby highlighting the importance of conducting prenatal diagnostics in such instances. Another interesting finding in our cohort was the presence of 33 cases (9.8%, 33/338) exhibiting a familial history of CL/P, including relatives in the 1st-degree or 2nd-degree in their families, and the majority of cases were classified as isolated CL/P (93.9%, 31/33). In these cases, only one instance of trisomy 18 was detected (case 8), and we hypothesize that this may be associated with advanced maternal age. A previous study highlighted a robust correlation between orofacial clefts and familial history [20], which is associated with an elevated risk of cleft occurrence within the family unit [21].

In our study, we identified three cases with simultaneous microdeletions and microduplications. The results indicated the presence of a balanced translocation variant in Case 9's mother, with the specific karyotype result being 46XX,t(5;21)(q31.1;q22.3). Ultimately, the family decided to undergo third-generation assisted reproductive technology (ART) during their next pregnancy, thereby effecting primary prevention [22]. This situation emphasizes the importance of understanding the limitations of CMA, as it cannot detect chromosomal balanced translocations [23, 24]. When CMA suggests the simultaneous presence of microdeletions and microduplications, alongside a family history of spontaneous abortion, attention should be given to the presence of chromosomal balanced translocations and further parental chromosomal karyotyping should be conducted. Additionally, considering the increased incidence of epigenetic and imprinting defects associated with ART, conducting long-term follow-up studies on children conceived through ART is crucial for a comprehensive understanding of potential epigenetic risks.

The predominant coexisting major anomaly identified in conjunction with oral clefts was cardiopathy, present in 30.3% (10/33) of cases. The close association between CL/P and congenital cardiovascular defects is not surprising considering the mechanisms of early embryonic development [25, 26]. The aortic arches of the primitive heart, indeed, encircle the pharyngeal arches, which subsequently undergo morphogenesis to form the facial structures [23, 27]. This also suggests that careful screening of the cardiac development in fetuses with CL/P should be conducted during the prenatal and postnatal periods to promptly identify any abnormal cardiovascular development in addition to further midline anomalies [28–30].

In the majority of cases, the outcome of pregnancy is largely influenced by the presence of combined malformations detected by prenatal ultrasound and the results of chromosomal analysis. Parents often opt for pregnancy termination in cases where a prenatally diagnosed pathogenic CNV carries a poor prognosis, such as the 4p16.3 deletion syndrome. Similarly, when subsequent ultrasound assessments reveal worsening conditions for fetuses with VUS, parents frequently choose TOP. Conversely, a negative result obtained through CMA can enhance parents' confidence in continuing the pregnancy. A notable distinction was observed between the rates of pregnancy termination in cases with clinically significant variants detected through genetic testing and those with negative test results (28/29, 96.6% vs. 103/295, 34.9%, *p* < 0.001). The findings from genetic testing in fetuses diagnosed with CL/P appear to have an impact on parental decision-making. Among cases where clinically significant variants were detected via genetic diagnosis, a small proportion of patients opted for pregnancy continuation following genetic counseling. Conversely, the majority elected for pregnancy termination due to potential postnatal phenotypic manifestations and unfavorable prognosis. Consequently, it is essential to conduct genetic testing in patients who receive a prenatal ultrasound diagnosis of fetal CL/P. This approach equips physicians and parents with additional insights regarding potential postnatal phenotypes, which in turn, aid in informed decision-making regarding pregnancy outcomes and post-birth management [31].

This study is subject to several limitations. Firstly, it should be noted that this study is retrospective, and as a consequence, certain critical parameters, such as parental examination, are absent from the results. Second, although our study included both singleton and twin pregnancies in the prenatal investigation of CL/P, as well as cases with a family history of CL/P in the fetus, the number of cases in these two groups was relatively limited. This limitation hampers our ability to draw conclusive inferences. We aim to gather additional cases of twin pregnancies to gain further insight into potential differences between them. Furthermore, it is worth noting that CL/P may be associated with a single gene, but we did not conduct further examinations such as WES.

## Conclusions

This study represents a comprehensive prenatal investigation utilizing CMA to conduct an intricate molecular examination of fetal CL/P cases within a Chinese population. The detection of fetal CL/P via ultrasound may serve as a diagnostic clue for certain syndromes, and the presence of ultrasound abnormalities appears to be associated with an elevated likelihood of CMA findings in singleton and twin pregnancies, as well as in cases of isolated and non-isolated CL/P. Consequently, in instances where fetal CL/P is identified through prenatal ultrasound, specific attention should be directed toward assessing potential co-occurring structural abnormalities and pursuing genetic testing to exclude the presence of genetic disorders. Such comprehensive evaluations may contribute to the enhancement of diagnostic precision and the provision of informed counseling in the context of prenatal CL/P identification.

### Supplementary Information

Supplementary Material 1: Supplementary Table 1 summarize the prenatal ultrasound phenotypes and pregnancy outcomes for the CL/P fetuses with VUS

## Data Availability

The data that support the findings of this study are not publicly available as the information contained could compromise the privacy of research participants. Further inquiries can be directed to the corresponding author.

## Abbreviations

CNVs: Copy number variants
CMA: Chromosome microarray analysis
QF-PCR: Quantitative fluorescence polymerase chain
CLP: Cleft lip and palate
CP: Cleft palate
UPD: Uniparental disomy
GA: Gestational age
MA: Maternal age
mos: Mosaicism
TOP: Termination of pregnancy
PRUV: Persistent right umbilical vein
CM: Low-lying conus medullaris
CTGA: Complete transposition of great arteries
AVSD: Atrioventricular septal defect
NT: Nuchal translucency
SUA: Single umbilical artery
DORV: Double outlet right ventricle
CH: Cystic hygroma
CSVs: Clinically significant variants
VUS: Variants of unknown significance
WES: Whole-exome sequencing
WGS: Whole-genome sequencing
ART: Assisted reproductive technology

## References

[1] Dixon MJ, Marazita ML, Beaty TH, Murray JC. Cleft lip and palate: understanding genetic and environmental influences. Nat Rev Genet. 2011;12(3):167–78. 21331089 10.1038/nrg2933 PMC3086810

[2] Group IW. Prevalence at birth of cleft lip with or without cleft palate: data from the International Perinatal Database of Typical Oral Clefts (IPDTOC). Cleft Palate Craniofac J. 2011;48(1):66–81. 20507242 10.1597/09-217

[3] Martelli DRB, Machado RA, Swerts MSO, Rodrigues LAM, de Aquino SN, Júnior HM. Non sindromic cleft lip and palate: relationship between sex and clinical extension. Braz J Otorhinolaryngol. 2012;78(5):116–20. 23108830 10.5935/1808-8694.20120018 PMC9450739

[4] Mossey P, Little J. Addressing the challenges of cleft lip and palate research in India. Indian J Plast Surg. 2009;42(S 01):S9–S18. 19884687 10.4103/0970-0358.57182 PMC2825065

[5] Wilhelm L, Borgers H. The ‘equals sign’: a novel marker in the diagnosis of fetal isolated cleft palate. Ultrasound Obstet Gynecol. 2010;36(4):439–44. 20521240 10.1002/uog.7704

[6] Merritt L. Part 1. Understanding the embryology and genetics of cleft lip and palate. Adv Neonatal Care. 2005;5(2):64–71. 15806447 10.1016/j.adnc.2004.12.006

[7] Armour CM, Dougan SD, Brock J-A, et al. Practice guideline: joint CCMG-SOGC recommendations for the use of chromosomal microarray analysis for prenatal diagnosis and assessment of fetal loss in Canada. J Med Genet. 2018;55(4):215–21. 29496978 10.1136/jmedgenet-2017-105013 PMC5869456

[8] Dugoff L, Norton ME, Kuller JA, Medicine SfM-F. The use of chromosomal microarray for prenatal diagnosis. Am J Obstet Gynecol. 2016;215(4):B2–B9. 27427470 10.1016/j.ajog.2016.07.016

[9] Shaffer LG, Rosenfeld JA, Dabell MP, et al. Detection rates of clinically significant genomic alterations by microarray analysis for specific anomalies detected by ultrasound. Prenat Diagn. 2012;32(10):986–95. 22847778 10.1002/pd.3943 PMC3509216

[10] Conte F, Oti M, Dixon J, Carels CE, Rubini M, Zhou H. Systematic analysis of copy number variants of a large cohort of orofacial cleft patients identifies candidate genes for orofacial clefts. Hum Genet. 2016;135:41–59. 26561393 10.1007/s00439-015-1606-x PMC4698300

[11] Li YY, Tse WT, Kong CW, Wong NK, Leung TY, Choy KW, To WW, Cao Y. Prenatal diagnosis and pregnancy outcomes of fetuses with orofacial cleft: A retrospective cohort study in two centres in Hong Kong. Cleft Palate Craniofac J. 2024;61(3):391–9. 36128746 10.1177/10556656221128436

[12] Maarse W, Berge S, Pistorius L, et al. Diagnostic accuracy of transabdominal ultrasound in detecting prenatal cleft lip and palate: a systematic review. Ultrasound in Obstetrics and Gynecology: The Official Journal of the International Society of Ultrasound in Obstetrics and Gynecology. 2010;35(4):495–502. 20235140 10.1002/uog.7472

[13] Wong HS, Pringle KC. Ultrasound imaging of the fetal palate. In: Ultrasound Imaging-Medical Applications. IntechOpen; 2011.

[14] Liao C, Fu F, Li R, et al. Implementation of high-resolution SNP arrays in the investigation of fetuses with ultrasound malformations: 5 years of clinical experience. Clin Genet. 2014;86(3):264–9. 24000829 10.1111/cge.12271

[15] Cao Y, Li Z, Rosenfeld JA, et al. Contribution of genomic copy-number variations in prenatal oral clefts: a multicenter cohort study. Genet Med. 2016;18(10):1052–5. 26913922 10.1038/gim.2015.216

[16] Schierz IA, Serra G, Antona V, Persico I, Corsello G, Piro E. Infant developmental profile of Crisponi syndrome due to compound heterozygosity for CRLF1 deletion. Clin Dysmorphol. 2020;29(3):141–3. 32433043 10.1097/MCD.0000000000000325

[17] Nasreddine G, El Hajj J, Ghassibe-Sabbagh M. Orofacial clefts embryology, classification, epidemiology, and genetics. Mutation Research/Reviews in Mutation Research. 2021;787: 108373. 34083042 10.1016/j.mrrev.2021.108373

[18] Saleem K, Zaib T, Sun W, Fu S. Assessment of candidate genes and genetic heterogeneity in human non syndromic orofacial clefts specifically non syndromic cleft lip with or without palate. Heliyon. 2019;5(12): e03019. 31886431 10.1016/j.heliyon.2019.e03019 PMC6921104

[19] Shkoukani MA, Chen M, Vong A. Cleft lip–a comprehensive review. Front Pediatr. 2013;1:53. 24400297 10.3389/fped.2013.00053 PMC3873527

[20] Figueiredo JC, Ly S, Magee KS, et al. Parental risk factors for oral clefts among Central Africans, Southeast Asians, and Central Americans. Birth Defects Res A. 2015;103(10):863–79. 10.1002/bdra.23417 PMC5049483 26466527

[21] Mbuyi-Musanzayi S, Kayembe TJ, Kashal MK, et al. Non-syndromic cleft lip and/or cleft palate: Epidemiology and risk factors in Lubumbashi (DR Congo), a case-control study. Journal of Cranio-Maxillofacial Surgery. 2018;46(7):1051–8. 29802056 10.1016/j.jcms.2018.05.006

[22] Serra G, Antona V, D’Alessandro MM, Maggio MC, Verde V, Corsello G. Novel SCNN1A gene splicing-site mutation causing autosomal recessive pseudohypoaldosteronism type 1 (PHA1) in two Italian patients belonging to the same small town. Ital J Pediatr. 2021;47(1):138. 34134742 10.1186/s13052-021-01080-x PMC8207710

[23] Serra G, Felice S, Antona V, Di Pace MR, Giuffrè M, Piro E, Corsello G. Cardio-facio-cutaneous syndrome and gastrointestinal defects: report on a newborn with 19p13. 3 deletion including the MAP 2 K2 gene. Ital J Pediatr. 2022;48(1):65. 35509048 10.1186/s13052-022-01241-6 PMC9069788

[24] Serra G, Antona V, Giuffrè M, Piro E, Salerno S, Schierz IA, Corsello G. Interstitial deletions of chromosome 1p: novel 1p31. 3p22. 2 microdeletion in a newborn with craniosynostosis, coloboma and cleft palate, and review of the genomic and phenotypic profiles. Ital J Pediatr. 2022;48(1):38. 35246213 10.1186/s13052-022-01232-7 PMC8896361

[25] Genisca AE, Frías JL, Broussard CS, et al. Orofacial clefts in the national birth defects prevention study, 1997–2004. Am J Med Genet A. 2009;149(6):1149–58. 10.1002/ajmg.a.32854 PMC3111146 19441124

[26] Piro E, Serra G, Giuffrè M, Schierz IA, Corsello G. 2q13 microdeletion syndrome: report on a newborn with additional features expanding the phenotype. Clinical Case Reports. 2021;9(6):e04289.

[27] Piccione M, Serra G, Consiglio V, Di Fiore A, Cavani S, Grasso M, Malacarne M, Pierluigi M, Viaggi C, Corsello G. 14q13. 1‐21.1 deletion encompassing the HPE8 locus in an adolescent with intellectual disability and bilateral microphthalmia, but without holoprosencephaly. Am J Med Genet A. 2012;158(6):1427–33. 10.1002/ajmg.a.35334 22581785

[28] Serra G, Antona V, Schierz M, Vecchio D, Piro E, Corsello G. Esophageal atresia and Beckwith-Wiedemann syndrome in one of the naturally conceived discordant newborn twins: first report. Clinical Case Reports. 2018;6(2):399. 29445485 10.1002/ccr3.1103 PMC5799623

[29] Piro E, Serra G, Schierz IA, Giuffrè M, Corsello G. Neonatal ten-year retrospective study on neural tube defects in a second level University Hospital. Ital J Pediatr. 2020;46:1–6. 32448340 10.1186/s13052-020-00836-1 PMC7247239

[30] Serra G, Giambrone C, Antona V, Cardella F, Carta M, Cimador M, Corsello G, Giuffrè M, Insinga V, Maggio MC, Pensabene M. Congenital hypopituitarism and multiple midline defects in a newborn with non-familial Cat Eye syndrome. Ital J Pediatr. 2022;48(1):170. 36076277 10.1186/s13052-022-01365-9 PMC9461219

[31] Serra G, Giuffrè M, Piro E, Corsello G. The social role of pediatrics in the past and present times. Ital J Pediatr. 2021;47:1. 34922600 10.1186/s13052-021-01190-6 PMC8684095

