# Supplementary material for: Association of prenatal Cleft Lip and Palate ultrasound abnormalities with copy number variants at a single Chinese tertiary center
Source: Ital J Pediatr. 2024 Aug 21;50:152. doi: 10.1186/s13052-024-01720-y (PMC11337895; doi:10.1186/s13052-024-01720-y)
Supplement: Supplementary file 1 — Supplementary Material 1: Supplementary Table 1 summarize the prenatal ultrasound phenotypes and pregnancy outcomes for the CL/P fetuses with VUS [file 13052_2024_1720_MOESM1_ESM.docx]

**Supplementary Table 1 Clinical and chromosomal characteristics of the 18 cases with VUS detected.**

| **Case** | **MA**  **(years)** | **GA**  **(weeks)** | **Ultrasound Findings** | **CMA Results** | **Type of CNV** | **Size** | **Outcome** |  |
| --- | --- | --- | --- | --- | --- | --- | --- | --- |
| 1 | 27.4 | 24.7 | Isolated CL | 18q22.3(72189157-72408358)×3 | Duplication | 219Kb | Live birth | |
| 2 | 37.6 | 24 | Isolated CL | 1p32.2(56784082-57291371)×3 | Duplication | 507Kb | Live birth | |
| 3 | 26.5 | 25.1 | Isolated CP | 13q12.12(23519916-24941516)×1 | Deletion | 1.42Mb | TOP | |
| 4 | 27.3 | 24.3 | Isolated CLP | 2q21.1(131,979,429-132,280,823)×4 | Duplication | 301Kb | Live birth | |
| 5 | 31.3 | 12.3 | Isolated CLP | 10q21.2(61962260-62294817)×3 | Duplication | 333Kb | TOP | |
| 6 | 21.0 | 24.4 | Isolated CLP | 4q22.1(92268538-93163393)×3 | Duplication | 895Kb | Live birth | |
| 7 | 33.9 | 23.1 | Isolated CLP | 18q21.31(54921857-55431046)×3 | Duplication | 509Kb | TOP | |
| 8 | 23.6 | 24.4 | Isolated CLP | 2q35(217,140,460-217,456,567)×3 | Duplication | 316Kb | Lost follow | |
| 9 | 33.1 | 24.6 | Isolated CLP | 7q11.21 (64623362-65148399)×1  Xp22.33 1932944-2270485 | Deletion  Duplication | 525Kb  338Kb | TOP | |
| 10^#^ | 33.6 | 26.0 | Isolated CLP | 7q22.1(102036215-102333120)×1 | Deletion | 297Kb | Live birth | |
| 11 | 23.4 | 26.6 | Isolated CLP | Xp22.33(168,551-542,709)×3 | Duplication | 374Kb | TOP | |
| 12 | 38.3 | 27.7 | Isolated CLP | 7p22.1p21.3(7080073-7516124)×3 | Duplication | 436Kb | Live birth | |
| 13 | 31 | 22.1 | Isolated CLP | 10q21.1(57098858-57328573)×1 | Deletion | 230Kb | Lost follow | |
| 14 | 27.2 | 25.4 | Isolated CLP | 1p34.1(46153071-46356402)×3 | Duplication | 203Kb | Lost follow | |
| 15 | 21.8 | 24.4 | Isolated CLP | 10q11.22q11.23(48117189-51110407)×3 | Duplication | 2.99Mb | Live birth | |
| 16 | 24.8 | 22.1 | Isolated CLP | 5q11.2(55160853-55872948)×3 | Duplication | 712Kb | Live birth | |
| 17 | 24.0 | 31.3 | CLP; duplex kidney; ureterectasis | 5q35.3(178709173-178931310)×3 | Duplication | 222Kb | TOP | |
| 18 | 27.5 | 24.3 | CLP; VSD | 2q23.2(149992405-150306499)×3 | Duplication | 314Kb | TOP | |

#: one of twin fetuses; CLP: cleft lip and palate; CL: cleft lip; CP: cleft palate; GA: gestational age; MA: maternal age; CNVs: copy number variations; TOP: termination of pregnancy; VSD: ventricular septal defect
